# 

**Published:** 1887-11

**Authors:** 


					﻿Vol. VII.
NOVEMBER, 1887.
No. 11.
THE
HOMEOPATHIC pHY^lClA|3,
A MONTHLY JOURNAL OF MEDICAL SCIENCE.
‘‘ He is a freeman whom truth makes free, and all are slaves beside.”
“Seek the Truth: come whence it may, cost what it will.'’
EDITED BY
Edmund j. lee, m. d.,
AND
WALTER ME. JAMES, ME. D.
PHILADELPHIA :
No. 1183 SPRTJCH STREET.
asrcHJTXr yos-k:
A. L. CHATTERTON A COMPANY, PUBLISHER’S AGENTS.
LONDON:
ALFRED HEATH A CO., Sole Agents fob Great Britain,
114 Ebury Street, Eaton Square.
Yearly Subscription, $2.50, invariably in advance. Single numbers, 25 cts.
Entered at the Philadelphia Port-Office as Second-Class Mail Matter.
				

